# Lack of professional recognition: main reason for stress in military police

**DOI:** 10.47626/1679-4435-2022-749

**Published:** 2023-02-03

**Authors:** Thalyta Brito Rafael dos Santos, Evanice Avelino de Souza, Felipe Rocha Alves

**Affiliations:** 1 Educação Física, Faculdade Terra Nordeste, Caucaia, CE, Brazil; 2 Departamento de Ciências Médicas, Universidade Federal do Ceará, Fortaleza, CE, Brazil

**Keywords:** occupational stress, police, mental health, cross-sectional studies, work., estresse ocupacional, polícia, saúde mental, estudos transversais, trabalho.

## Abstract

**Introduction:**

Military police officers play a crucial role in contemporary society, which is marked by the increase in criminality. Therefore, these professionals are constantly under pressure, both socially and professionally, so occupational stress is something present in their routine.

**Objectives:**

To investigate the stress levels of military police officers in the municipality of Fortaleza and its metropolitan region.

**Methods:**

This was a cross-sectional, quantitative study, conducted with 325 military police officers (53.1% men; 20> 51 years old) who belonged to military police battalions. The Police Stress Questionnaire was used to identify the stress level, following the Likert scale from 1 to 7; the higher the score, the higher the stress level.

**Results:**

The results indicated that the lack of professional recognition is the main stress factor among military police officers (Median = 7.00). Other items were relevant to the quality of life of these professionals, which are: “risks of injuries or wounds resulting from the profession”, “working on days off”, “lack of human resources”, “excessive bureaucracy in the police service”, “ having the perception that we are pressured to give up free time ”,“ lawsuits resulting from police service,” “going to court, relationship with the judicial actors, ” and “use of inadequate equipment for the service,” respectively (Median = 6. 00).

**Conclusions:**

The stress of these professionals is organizational in nature and comes from factors that transcend the violence with which they deal.

## Abstract (pt)

**Introdução:**

O policial militar desempenha uma função de suma relevância na sociedade contemporânea, a qual é marcada pelo avanço da criminalidade. Desse modo, esse profissional se encontra constantemente sob pressão, tanto social quanto trabalhista, de maneira que o estresse ocupacional é algo presente em sua rotina.

**Objetivos:**

Investigar os níveis de estresse em policiais militares da cidade de Fortaleza e região metropolitana.

**Métodos:**

Trata-se de um estudo transversal, de natureza quantitativa, realizado com 325 policiais militares, sendo (53,1% do sexo masculino; 20 > 51 anos de idade) pertencentes aos batalhões de policiamento militar. O questionário Police Stress Questionnaire foi utilizado para identificar o nível de estresse, seguindo a escala Likert de 1 a 7 - quanto maior a pontuação, maior o nível de estresse.

**Resultados:**

Os resultados indicaram que a falta de reconhecimento profissional é o principal fator de estresse entre os policiais militares (mediana = 7,00). Outros itens foram relevantes na qualidade de vida desses profissionais, são eles: “riscos de lesões ou ferimentos resultantes da profissão”, “trabalhar em dias de folga”, “falta de recursos humanos”, “excesso de burocracia no serviço policial”, “ter a percepção que somos pressionados a abdicar o tempo livre”, “processos de justiça decorrentes do serviço policial”, “idas ao tribunal, relação com os intervenientes judiciais” e “uso de equipamentos inadequado para o serviço” respectivamente (mediana = 6,00).

**Conclusões:**

O estresse desses profissionais é de cunho organizacional e provém de fatores que transcendem à violência com a qual lidam.

## INTRODUCTION

The International Stress Management Association (ISMA)^^1^^ indicated Brazil as the country with the second highest level of stress in the world. According to the research, the main reason for stress is work, considering the very long working hours, the lack of time for personal activities, and the harmful corporate environment.

According to the Ministry of Health,^^2^^ professions with high levels of occupational stress, responsibilities, and risks are more likely to cause mental illness, which results from the stress experienced in the work environment. Thus, among the most stressful professions are, respectively: doctors, nurses, teachers, police officers, and journalists.

According to the Association of Military Police and Firemen of the State of Ceará,^^3^^ psychological disorders such as occupational stress and depression are the main factors that lead individuals to suicide. It is noteworthy that in Brazil alone, in 2018, a total of 53 military police officers committed suicide, and in 2019, in Ceará alone, six officers ended up taking their own lives.

The military police profession is considered one of the most stressful in the world, due to issues such as direct contact with violence, long working hours, as well as low salaries, and inflexible scales.^^4^^

In this sense, the stress acquired as a result of these factors can contribute to the emergence of mental disorders, such as anxiety, panic syndrome, burnout syndrome, and depression, frequent diseases in this class of workers.^^5^^

According to the Brazilian Public Security Forum (2018), Ceará was the Northeastern state with the highest number of police officers killed on duty, and the capital city Fortaleza is considered the second most violent city in Brazil and the ninth most violent in the world, with 83.48 homicides per 100.000 inhabitants.^^6^^

Data like these can generate fear and insecurity, and raise stress levels even higher, given the importance of this issue in the context of public safety. The present study aimed to investigate the levels of stress among military police officers in the city of Fortaleza and its metropolitan region, to understand which factors corroborate the growing evolution of dissatisfied and overwhelmed professionals.

## METHODS

This is a quantitative, cross-sectional study conducted in the period from May to June 2019 in four battalions of the military police force in the city of Fortaleza, state of Ceará, and its metropolitan region, including the Police Battalion of the External Guard of Detention Centers (Batalhão de Policiamento da Guarda Externa de Presídios, BPGEP), the Shock Police Command (Comando de Policiamento de Choque, CPCHOQUE), the Police Command of Intensive and Ostensive Action Patrols (Comando de Policiamento de Ronda de Ações Intensivas e Ostensivas, CPRAIO), and the 8th and 12th General Ostensive Police (8° e 12° Policiamento Ostensivo Geral, POG).

The employees were approached during working hours, invited to participate voluntarily, and informed about the study and the expected benefits, assuring anonymity.

The study was referred to the Research Ethics Committee of the Faculdade Terra Nordeste (FATENE) and approved under opinion number 219240194500008136. The research followed the norms for research with human beings of resolutions 466/2012 and 510/2016.

According to the organization of each battalion, we were informed that there was around 1,035 military personnel, however, 657 were on leave for different reasons. Thus, the 378 active professionals were invited to participate in the research.

**Figure 1:**
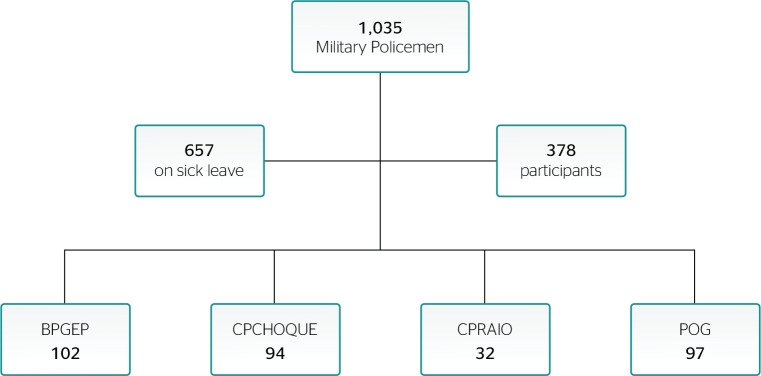
Description of the population and sample of the present study. BPGEP = Batalhão de Policiamento da Guarda Externa de Presídios (Police Battalion in charge of the External Guard of Detention Centers); CPCHOQUE = Comando de Policiamento de Choque (Shock Police Command); CPRAIO = Comando de Policiamento de Ronda de Ações Intensivas e Ostensivas (Police Command of Intensive and Ostensive Action Patrols); POG = 8° e 12° Policiamento Ostensivo Geral (8th and 12th General Ostensive Police).

For the sample estimate, the exclusion criteria were: the absence of military police officers at the time of collection, incorrect answers to the questionnaire, and failure to sign the Informed Consent Form (ICF). Thus, 18 policemen answered the questionnaires incorrectly, 25 refused to participate claiming to be off duty, and 10 did not sign the ICF (Figure 1).

The instrument used to assess stress levels was the Police Stress Questionnaire (PSQ), a 40-item instrument composed of two subscales that measure organizational and operational stressors in the context of police work, using 7-point scales (Likert type), ranging from 1 (no stress) to 7 (extreme stress). The PSQ was developed as an alternative to general job stress scales to reflect the specific stressors of police officers^^7^^ that have already been used in Brazil.^^8^^

The sociodemographic variables included sex, age, time of service, work unit, and practice of physical activity in childhood and adolescence.

Initially, it was identified that the data did not present normality by the Kolmogorov-Smirnov test, and a descriptive analysis was performed using the median and interquartile range for the description of the results, using SPSS, version 21.0.

## RESULTS

The present study included a sample of 325 policemen (61.5% male) that make up the BPGEP, the CPCHOQUE, the CPRAIO, and the 8th and 12th POG Commands.

We observed a prevalence of 135 (47.2%) military personnel between 31 and 40 years of age, 87 (30.3%) who had been working for 5 to 10 years, and 102 (31.7) who worked in the BPGEP unit. The other characteristics are described in Table 1.

**Table 1:** General characteristics of the military police battalion of the state of Ceará - Fortaleza and its metropolitan region 2019

Variables	n	%
Sex		
Women	125	38.5
Men	200	61.5
Age (years)		
20-30	67	23.4
31-40	135	47.2
41-50	76	26.6
> 50	8	2.8
Time of service (years)		
1st quartile (up to 5)	76	26.5
2nd quartile (5 to 10)	87	30.3
3rd quartile (10 to 18)	59	20.6
4th quartile (18)	65	22.6
Unit of Work		
12th BPM	97	30.1
BPGEP	102	31.7
CPRAIO	32	9.9
CPCHOQUE	91	28.3

BPGEP = Police Battalion in charge of the External Guard of Detention Centers; BPM = Military Police Battalion; CPCHOQUE = Shock Police Command; CPRAIO = Police Command of Intensive and Ostensive Action Patrols.

A higher level of occupational stress was observed in the questions regarding the risks of injury or harm resulting from the profession [6.00 (4.00-7.00)] and working on days off (e.g., going to court, events in the community, etc.) [6.00 (4.00-7.00)], such as displayed in Table 2.

**Table 2:** Level of occupational stress in the military police battalion of the state of Ceará - Fortaleza and its metropolitan area 2019

Variables	Median	IQR
1. Working in shifts	4.00	2.00-5.00
2. Working alone at night	3.00	1.00-6.75
3. Working overtime	5.00	3.00-7.00
4. Risks of injury or occupational harm	6.00	4.00-7.00
5. Working on days off, e.g. going to court, events in the community, etc.	6.00	4.00-7.00
6. Traumatic events, e.g. death, injury, domestic violence, etc.	5.00	3.00-7.00
7. Managing social life outside of work	3.00	2.00-5.00
8. Lack of time for friends and family	4.00	3.00-6.00
9. Preparing notices	3.00	1.00-4.00
10. Maintaining healthy eating habits at work	4.00	2.00-6.00
11. Lack of time to maintain good physical condition	4.00	2.50-6.00
12. Fatigue (resulting from shift work, overtime, etc.)	5.00	3.00-7.00
13. Health problems arising from work, e.g. back pain, etc.	5.00	3.00-7.00
14. Lack of understanding of people regarding our availability	4.00	2.00-6.00
15. Difficulty in creating friendships outside the professional sphere	3.00	1.00-500
16. Impact of my actions on society inside and outside the service	4.00	2.00-6.00
17. The image that society has of me as a military servant	4.00	2.00-6.00
18. Constraint in my personal life derived from service	4.00	2.00-5.00
19. The feeling of being permanently on duty	5.00	2.00-7.00
20. Family and friends, who feel the negative effects of my profession	4.00	2.00-6.00

IQR = interquartile range.

Regarding the issues related to the recognition of the professional commitment of the workers under analysis, it was noticed that there is a high level of stress due to the lack of this recognition, the lack of perception of the abdication that public servants have given the function they perform for the Government, as shown in Table 3.

**Table 3:** The occupational stress level of the military policing battalion of the state of Ceará - Fortaleza and its metropolitan area 2019

Variables	Median	IQR
21. Getting along with colleagues	2.00	1.00-4.00
22. Feeling that the rules do not apply, that they differ from person to person	5.00	3.00-7.00
23. Feeling that we are permanently evaluated	5.00	3.00-7.00
24. Excessive workload	5.00	3.00-7.00
25. Constant changes in legislation, internal organization norms	5.00	3.00-700
26. Lack of human resources	6.00	4.00-7.00
27. Excessive bureaucracy in the police service	6.00	4.00-7.00
28. Excessive use of electronic means	4.00	2.00-5.00
29. Lack of training and equipment	5.00	3.00-7.00
30. Having the perception that we are pressured to give up free time	6.00	400-7.00
31. Dealing with hierarchical superiors	4.00	2.0-6.00
32. Leadership style, unconscious command	4.00	2.25-600
33. Lack of material and financial resources	5.00	3.00-7.00
34. Poor distribution of responsibilities among the military	5.00	3.00-7.00
35. Feeling contempt from colleagues when we are sick	5.00	3.00-7.00
36. The chain of command overemphasizes the negative aspects of police service	4.00	3.00-6.00
37. Lawsuits arising from the police service	6.00	4.00-7.00
38. Going to court, relationship with court actors	6.00	4.00-7.00
39. Lack of recognition of our effort	7.00	5.00-7.00
40. Inadequate use of equipment for the service	6.00	4.00-7.00

IQR = interquartile range.

## DISCUSSION

The main indicator of stress in the sample is the lack of recognition for the effort made. These results corroborate other studies conducted with Brazilian police officers.^^9^-^14^^ This factor may be related to aspects such as low salaries, since we observed a considerable salary disadvantage in comparison to the amount perceived by officers working for corporations in other Brazilian states. Low salaries represent a lack of recognition of the professional, especially when the duties of the service are the same.^^10^^

Another aspect that causes the feeling of frustration, uselessness, and unproductivity is the working conditions, which are related to the lack of equipment and the scarcity of police facilities and human resources during the service.^^10^,^15^^

Besides the need for investment and retribution so that the professional feels valued, it is crucial to highlight the actions developed by the corporation for the community, such as the Educational Program of Drug Resistance (Proerd), the preventive anti-drug action for children and teenagers conducted in schools; ecotherapy, a therapeutic method that helps in the treatment of disabled children; and the disclosure of statistics showing all the approaches, arrests, and confiscations made by the military police in a given region^^16^^ on the government’s official website.

Another stressor is working on days off. However, it is noteworthy that other studies^^17^-^20^^ conducted in the South and Southeast regions of Brazil did not obtain results similar to those of the present study. This divergence is justified by the greater economic development of the aforementioned regions, when compared to the Northeast region.

It is considered that this economic difference causes a search for extra services as a way to complement and/or increase the income of military police officers.^^21^^ The extra work, besides providing an additional salary, can cause physical and psychological harm to the individual’s health.^^22^^

Working overtime, having more than two jobs, or working for more than 12 hours negatively interferes with a human being’s quality of life. In this sense, one possibility to minimize the impact of stress related to working on days off would be to adjust the salaries of police officers, so that the differences between the regions of the country would not be perceived or would not be so evident, and the wages would be understood based on the number of inhabitants, in addition to providing a housing plan and a health plan for the officers, in appreciation.^^23^^

The risk of injury was another factor considered stressful. Injuries can be the result of several causes, such as immobilizing individuals, chasing and overcoming obstacles, among others. Moreover, besides their own body weight, military policemen wear uniforms, weapons, ammunition clips, ballistic vests, and handcuffs, which imply the need for more physical effort, making them more prone injury.^^24^-^26^^

Studies have pointed out^^27^,^28^^ that for better functionality and a more favorable performance during actions, it is necessary to balance work and physical fitness. Another study verified that military police officers with higher levels of physical fitness are less likely to suffer sprains, low back pain, and chronic pain. Thus, some changes, such as the organization of rest time, job rotation, and the administration of a training routine within the institutions, should considerably improve the impacts of repetitive strain.

The lack of human resources was also considered a stressful factor. In Brazil, there is a great discrepancy regarding the number of police officers per inhabitant, mainly due to the current social dynamics, with the increase of tourists and events in public spaces, indicating the need for a larger number of military police officers. A study conducted^^30^^ in the city of Curitiba proposed a way to calculate the ideal number of officers and concluded that the establishment of this number per municipality is a perspective that needs attention since it is essential to preventive ostensive policing and the maintenance of public order.

Thus, it is very pertinent to apply measures already mentioned in the literature, such as the increase of vacancies in public competitions to join the military corporation; and the organization and elaboration of diagnoses of the Military Police, organizational situation, aiming at the importance of reorganizing and creating mechanisms capable of measuring the number of personnel per municipality, considering, mainly, the peculiarities of each place, the demographic evolution, and the criminal incidence.

Moreover, constant visits to the courts and participation in judicial proceedings were also perceived as stressful factors, results that corroborate the literature.^^13^^ The military police officer, for developing his work in criminal actions, such as robberies, and homicides, among others, ends up being necessary for the judicial proceedings, which implies frequently going to court on his days off, thus impacting his quality of life, since he gives up his time to rest in favor of society.^^9^,^14^,^15^^ Therefore, to improve the impact of stress concerning court visits, the ideal would be to count the hours the server spent in court and revert them into rest periods. As for court cases, specific and constant legal support is essential, so that the government can support and pay for the procedural costs until there is a final judgment.

Nevertheless, the present study also presented limitations. The first is related to the fact that the sample was drawn from only five units, so it prevented a larger number of participants. The second is related to the use of a cross-sectional design that did not allow us to indicate cause and effect relationships. However, the results found may serve for future interventions that aim to improve the levels of stress among military police officers in Fortaleza and its metropolitan region.

## CONCLUSIONS

The present study identified the lack of recognition as the greatest indicator of stress in police officers, once the military police officer has to deal directly with criminality in the search for the maintenance of public order. However, other reasons, such as working on days off, the risks of injury, the lack of human resources, the excess of bureaucracy, the invisibility of giving up free time, court visits, lawsuits, and the use of inadequate equipment, were also considered stress generating factors.

In this sense, to minimize the levels of stress, it is relevant that these professionals can understand the importance of mental health, and that they are provided with psychological counseling, aiming at the possibility of working out the emotional damage suffered by the intrinsic load of the function they perform, which permeates the field of violence, crime, among the other stressors brought up in this discussion.

Therefore, a more humane and dignified look at the military police officer is necessary, both from the State and from society. Society should welcome more humanely these professionals who daily seek the protection of all, and the State, as a promoter of the basic needs of the human being and as the employer of the public officer, once a fairer payment and the perception of a better recognized career will reduce the stress of these officers.
